# Married women’s decision making power on family planning use and associated factors in Mizan-Aman, South Ethiopia: a cross sectional study

**DOI:** 10.1186/s12905-016-0290-x

**Published:** 2016-03-08

**Authors:** Abeba Daniel Belay, Zelalem Birhanu Mengesha, Manay Kifle Woldegebriel, Yalemzewod Assefa Gelaw

**Affiliations:** Woman, Children and Youths Affairs Bureau, Bench Maji Zone, Mizan Aman, Ethiopia; Institute of Public Health, College of Medicine and Health Sciences University of Gondar, Gondar, Ethiopia

**Keywords:** Married women, Decision-making power, Family planning, Mizan-Aman City

## Abstract

**Background:**

Women’s use of family planning service is influenced by many factors, especially by their decision making power. A woman’s decision-making power, be it individual or decision made in collaboration with a partner, is the most important factor in the use of family planning in a household. The purpose of this study was to assess the impact of women’s decision making power on family planning use and its associated factors.

**Methods:**

A community-based cross-sectional study was conducted on married women in the child bearing age. The women who were living in Mizan city were selected using the simple random sampling method. Trained nurses collected the data by interview, using a structured and pre-tested questioner. Bivariable and multivariable binary logistic regression analysis was used to identify the associated factors, and the odds ratio with a 95 % CI was computed to assess the strength of the association. Collinearity was also assessed by looking at standard errors in the final fitted model.

**Result:**

Overall, more than two-thirds [67.2 %: 95 % CI (63–71 %)] of the married women were found to be more autonomous to decide family planning use. Secondary education [AOR: 9.04, 95 % CI: (4.50, 18.16)], government employment [AOR: 4.84, 95 % CI: (2.03, 11.52)], being wives of government employed spouses [AOR 2.71, 95 % CI: (1.24, 7.97)], having husbands with college or university education [AOR: 11.29, 95 % CI: (4.66, 27.35)], and being in the younger age [AOR: 0.27, 95 % CI :(0.09, 0.75)] were significantly associated with women’s decision-making power on family planning.

**Conclusions:**

In this study, women had a high decision making power in family planning use. Age category (34–44-years), formal education, and occupational status had effects on women’s decision making power. Promoting parental adult education and engaging women in out of house employment is essential to improve their decision making power in using family planning.

## Background

Family planning is a major issue for many developing countries where poor maternal and child health care services are practiced [1–4]. Even though the Ethiopian Government supports maternal health services and has gained some improvements in contraceptive use in the last few years, the total fertility rate per woman (4.1) and maternal mortality remain high (676 deaths per 100,000) [5], while family planning use remains low, especially in the rural settings (38.1 %) [6]. Previous isolated studies indicated the relationship between women’s decision making power and family planning use [7, 8]. The 2011 report of Ethiopian Demographic and Health Survey also confirmed that the proportion of family planning use was higher among women who decided individually without the collective involvement of their partners.

Independent decision making or decision making with communications with partners on family planning use has a substantial contribution to the improvement of maternal health [9]. Similarly, ensuring family planning access and allowing women to decide independently on the use of family planning is important in preventing unintended pregnancy [10].

Although women will be more benefited from their family planning use by achieving their human rights to health autonomy and decision making on family size [11], only less than one fourths of the women in Ethiopia are able to decide on family planning use by themselves [12].

Producing information on women’s decision making power in family planning use at the household level and identifying associated factors has a paramount importance. However, the existing information in Ethiopia on this issue is often very limited, making the planning, implementation, and evaluation of family planning activities difficult for program planners. Therefore, this study highlighted the relationship between women’s decision-making power, family planning use, and associated factors.

## Methods

### Study design, period, and setting

A community-based cross-sectional study design was applied from August 15 to 30/2013 in Mizan-Aman city administration which is located in Bench-Maji Zone, 227 Km south of Addis Ababa, the capital city of Ethiopia. The total population of the city administration in 2013 was 34,080 of whom 15,942 were women [13]. One general hospital, a health center, and about 30 private clinics provided family planning services in the city administration.

### Sample size determination and sampling techniques

The sample size of the study was computed by the single population proportion formula using the Epi Info™ version 3.5.3 Statcalc program considering: proportion(p) of independent decision-making on family planning use by urban married women (64 %) [7], level of significance (α = 0.05), worst acceptable values (60 or 68 %), and 10 % non-response rate. Finally, 608 was the final sample size of the study.

Married women in the age range of 15–49 years and lived at least for 6 months in the study area were selected using the simple random sampling techniques via a table of random generation. The list of the study population was obtained from health extension workers’ (the lowest health professionals working at health posts). Before data collection, a sampling frame was designed by numbering the list of house owners using the registration book.

### Data collection procedures and instruments

The primary data was collected by trained nures. A stractured and pretested questionarrie was used to collect the data. A half day training was given to the data collectors and supervisors on the objective, confidentiality of information, respondent rights, and the techniques of interview. Following the training, a pretest was administered (5 % of the sample size:30 married women) out of the study area before the actual data collection.

Information on socio-demographic characteristics, reproductive related history, knowledge about family planning, and women’s decision-making power were collected from married women. The collected data was checked for completeness and accuracy by the supervisors and the principal investigators on daily basis during the data collection process.

### Data management and analysis

The decision making power of women in family planning use was measured in relation to their ability to freely decide individually, discuss with their partners about family planning needs and choices using eight questions. A score of 1 was given if women decided independently or together by discussing the use of family planning, number of children, choice of family planning methods, when to give birth, where to get family planning service, how to seek reproductive health services with no oppositions about using/intending to use family planning. Zero (0) was scored by partners who decided independently. Then woman who scored below the mean were considered as having no decision making power, and those who scored greater or equal to the mean were considered as having good decision making power [7].

Data were entered using the Epi Info™ 3.5.3 and exported to the Statistical Package for Social Sciences (SPSS) version 20 for analysis and further cleaning purposes. Since the outcome variable was dichotomous, binary logistic regression models (both bivariable and multivariable) were used to identify associated factors. The Hosmer-Lemeshow goodness-of-fit statistic (0.67) was used to assess the fitness of the model. Odds ratios (Crude: COR and Adjusted: AOR) with 95 % confidence intervals (CI) were calculated to measure the strength of the association. In the multivariable analysis variables with a p-value of < 0.05 were considered as significant.

### Ethical consideration

This study was carried out after getting ethical approval from the Institutional Review Committee of the Institute of Public Health, the University of Gondar. Before the approval, the proposal was sent to reviewers to assure ethical issues. Finally, the Ethical Review Committee approved the oral consent on the ground that the research had no serious harm on the study participants. Before the interview, the interviewer fully explained the purpose of the study to each participant and obtained full verbal informed consent. To ensure confidentiality, no names were used in the questionnaire and in reporting the results of the study.

## Result

### Socio demographic characteristics of the respondents

A total of 567 married women within the reproductive age (with a response rate of 93.26 %) participated in the study. The mean age (SD) of the respondents was 29.53 (±6.79) years. The majority of the respondents were in the age group of 25–34 years. Three-fourths (79.4 %) of the women had attended primary school and above. Around 31 % were housewives (Table 1).

**Table 1:** Socio-demographic characteristics of married women’s decision making power in family planning use and associated factors in Mizan-Aman, South Ethiopia, 2013

Variables	Frequency (%)
Age category of women	
15–24	126 (22.2 %)
25–34	278 (49.0 %)
35–44	132 (23.3 %)
45–49	31 (5.5 %)
Ethnicity of women	
Bench	176 (31.0 %)
Amhara	120 (21.2 %)
Kefficho	110 (19.4 %)
Tigre	40 (7.1 %)
Oromo	48 (8.5 %)
Gurage	50 (8.8 %)
Others^a^	23 (4.1 %)
Religion of women	
Orthodox	197 (34.7 %)
Muslim	88 (15.5 %)
protestant	255 (45.0 %)
Others^b^	27 (4.8 %)
Occupation of women	
House wife	176 (31.0 %
Self employed	219 (38.6 %)
Governmental	172 (30.3 %)
Husbands occupation	
Not employed	81 (14.3 %)
Self employed	270 (47.6 %)
Governmental	216 (38.1 %)
Women educational status	
Can’t read and write	117 (20.6 %)
Primary education	207 (36.5 %)
Secondary education	162 (28.6 %)
College/university	81 (14.3 %)
Husbands educational status	
Can’t read and write	81 (14.3 %)
Primary education	161 (28.4 %)
Secondary education	170 (30.0 %)
College/university	155 (27.3 %)
Exposure to media	
Yes	490 (86.4 %)
No	77 (13.6 %)

Others^a^:-Wolayta, Yem, Dawro

Others^b^:-Catholic, Traditional

### Reproductive history and knowledge of respondents

The mean age at first marriage was 19.56 years with 2.72 SD. About 85 % of the women had given birth to one or more children and the majority (80.42 %) had desire for more children.

Around 98.8 % of the participants had heard about contraceptive and knew at least one method of family planning. The majority (95.2 %) desired to know more about family planning methods. Injectables (95.1 %) were the most used family planning methods. Fifty-eight percent of mothers had discussed family planning methods with their partners, and 36.2 % consulted health providers about the type of family planning and side effects (Table 2).

**Table 2:** Knowledge of married women’s decision making power in family planning use and associated factors in Mizan-Aman, South Ethiopia, 2013

Variables	Frequency (%)
Source of information	
HEWs	247 (43.6)
Radio	214 (37.7)
Television	377 (66.7)
Health centers	277 (48.9)
Formal Education	68 (12.0)
Places where to get FP services	
Hospital	404 (71.3)
Health center	432 (76.2)
Health post	141 (24.9)
Pharmacy	227 (40.0)
Clinics	239 (42.2)
Types of family planning methods	
Pills	425 (75.0)
IUCD	344 (60.7)
Inject able	539 (95.1)
Implant/Norplant	361 (63.7)
Female condom	93 (16.4)
Tuba ligation	136 (24.0)
Vasectomy	68 (12.0)
Male condom	294 (51.9)
Emergency contraceptive	169 (29.8)
Calendar method	181 (31.9)

### Women’s decision making power on family planning use

The overall proportion of women with a decision-making power was 67.2 % [95 % CI: (63–71 %).

### Factors associated with Women’s decision making power in family planning use

Women’s age, religion, educational status, occupation, exposure to media, partner’s educational status and occupation, number of living children, discussion with partner about family planning, and attitude towards family planning methods had *p* < 0.2 in the bivariate analysis and entered into multivariate analysis. Finally, in the multivariate analysis women’s age, educational status, occupation and partner’s educational status remained significantly associated with women’s decision making power in family planning use (Table 3).

**Table 3:** Factors associated with married women’s decision making power in family planning use and associated factors in Mizan-Aman, South Ethiopia, 2013

Explanatory variable	Decision making power		COR (95 % CI)	AOR (95 % CI)
	Yes	No		
Age				
15–24	95	31	1	1
25–34	192	86	2.38 (1.12–5.03)	0.41 (0.15,1.11)
35–44	79	53	1.72 (0.72–3.48)	0.26 (0.09, 0.74)*
45–49	15	16	3.26 (1.45–7.36)	0.57 (0.20, 1.63)
Women educational status				
Can’t read and write	29	88	1	1
Primary school	151	56	8.18 (4.86–13.76)	4.59 (2.49, 4.82)*
Secondary school	139	28	14.52 (8.09–26.06)	9.04 (4.50, 18.16)**
College/university	77	14	14.52 (7.12–29.61)	4.84 (2.03, 11.52)**
Husband educational status				
Can’t read and write	23	58	1	1
Primary school	76	85	2.25 (1.27–4.00)	1.18 (0.59, 2.35)
Secondary school	140	30	11.76 (6.30–21.95)	6.28 (3.01, 13.07)*
College/university	142	13	27.54 (13.07–58.05)	11.28 (4.65, 27.34)**
Women’s Occupation				
House wife	78	98	1	1
Self employed	157	62	3.18 (2.09–4.83)	1.88 (1.09, 3.24)*
Government employee	146	26	7.05 (4.22–11.77)	4.10 (2.11, 7.96)**
Exposure to media				
Have exposure	351	139	3.95 (2.40–6.51)	
No exposure	30	47	1	
Husband occupation				
Not employed	23	58	1	1
Self employed	174	96	4.57 (2.65–7.87)	1.47 (0.71, 3.03)
Government employee	184	32	14.50 (7.86–26.73)	2.7 (1.23, 5.94)*
Attitude towards FP methods				
Have favorable attitude	105	24	2.56 (1.58–4.16)*	
Have no favorable attitude	276	162	1	
Number of living children				
No children	68	21	1	
1–2	231	111	0.64 (0.37–1.10)	
3–4	64	38	0.52 (1.27–0.97)	
> 5	18	16	0.34 (0.15–0.79)*	

**P-value < 0.001, *p-value < 0.05

Young women in the age range of 34–44 years [AOR: 0.26, 95 % CI: 0.09, 0.47)] were 3.8 times more likely to have a decision-making power on family planning use as compared to older women. Women who had attended primary school [AOR: 4.59, 95 % CI: (2.49, 4.82)], secondary school [AOR: 9.04, 95 % CI: (4.50, 18.16)], and college/university [AOR: 4.84, 95 % CI (2.03, 11.52)] were more likely to have a decision-making power as compared to women unable to read and write. Similarly, women whose husbands had attended secondary school [AOR: 6.28, 95 % CI: (3.01, 13.07)] and college/university [AOR: 11.28, 95 % CI: (4.65–27.34)] were more likely to have a decision-making power as compared to their counter parts.

Self-employed [AOR: 1.88, 95 % CI: (1.09, 3.24)] and government employed women [AOR: 4.10, 95 % CI: (2.11, 7.96)] were more likely to have decision making power when compared to housewives. Similarly, women whose husbands were government employees were [AOR: 2.71, 95 % CI: (1.23–5.94)] 2.71 times more likely to have a decision making power than women who had unemployed husbands.

## Discussion

The empowerment of women has been reported to be a key to using family planning [14]. In developing countries most partners give inferior positions to women in all aspects of decision-making [7, 8, 14]. Consequently, women are either under collective decision-making with their partners and/or entirely rely on the male partner’s decision on issues that affect their contraception usage and reproductive life. In addition to family planning use, empowering women has an important role in the reduction of maternal and new born mortality by preventing unintended pregnancy and unsafe abortion [5, 15, 16]. In this study, the magnitude of women’s decision making power on family planning was 67.2 % (95 % CI: 63–71 %) which is consistent with a study done in Dawro Zone 64 %[7], but higher than the national report of Ethiopia (25 %) [5, 17]. This discrepancy might be due to the fact that this study considered only urban women in one district, whereas the national survey considered both urban and rural dwellers of at least more than one district, and it might also be due to the difference in educational status, and cultural norms of the women in the study settings. This study also confirms that women’s decision making power is consistent with studies done in India (68 %) [18], Malawi (28.75 %) [19] and Pakistan (28 %) [20].

This study found out that women’s employment status contributed to their decision making power in family planning use. Employed women are more likely to decide individually on family planning use which is in line with previous studies [17, 19, 21, 22] done in Ethiopia and other developing countries but negatively associated with studies done in southern Ethiopia [7]. This difference might be explained by the difference in job opportunities for urban and rural women.

Likewise, other previous studies [17, 19, 21–26] showed that the more educated the women and their partners, the more likely they were to decide on family planning use.

If a woman gets older and approaches menopause age, she may feel more confident to decide on family planning use individually and by discussing with her partner [16, 26, 27]. The findings of this study also suggest that there is a positive relationship between women’s age and their decision-making power on family planning use, which is in line with previous studies in Ethiopia [22, 23, 28]. Paradoxically, in the national study of Ethiopia 2011 [5], women’s decision-making power on family planning use decreased when they approached menopause. This discrepancy could be due to the difference in study settings, cultural values, and experience about family planning use.

This study has the following potential limitations. First, information on socio-demographic characteristics, knowledge, and attitudes towards family planning use intended to be gathered from husbands was obtained from the women. Second, the relationship between women’s autonomy and family planning use was measured by self-report, which is likely to be subjected to social desirability bias. Regarding participants, the study excluded women living in union, but not legally married. Moreover, attitude related to the reproductive history of the women was not supported with qualitative data.

## Conclusion

The magnitude of women’s decision making power on family planning among currently married reproductive age women was found to be high in this study. Factors, such as women’s and husbands’ primary and above education, age category (34–44 years), occupational status of women and their husbands were found to be statistically significantly related with the decision making power of women on family planning utilization. We recommend promoting parental adult education and engaging women on out of house employment.
